# The Association of Rural-Urban Inhabitation With Gastric Adenocarcinoma Mortality and Treatment: A Surveillance, Epidemiology, and End Results (SEER)-Based Study

**DOI:** 10.7759/cureus.18571

**Published:** 2021-10-07

**Authors:** Ahmed A Minhas, Zainab Fatima, Sai Karthik Kommineni, Zaid Ahmad, Sohail A Minhas

**Affiliations:** 1 Internal Medicine, East Tennessee State University Quillen College of Medicine, Johnson City, USA; 2 Medicine, Meharry Medical College, Nashville, USA; 3 Oncology, Baptist Memorial Hospital, Memphis, USA

**Keywords:** cancer mortality, public health, epidemiology, gastric cancer

## Abstract

Background

Gastric cancer is one of the most prevalent cancers in the world and the third most common cause of death from cancer. The diagnosis and treatment are often complex and require a multifaceted approach. Hence, appropriate and timely management is essential for better patient outcomes. Our aim was to determine if rural inhabitation affects the mortality of patients with gastric adenocarcinoma. If such an association exists, we propose to ascertain whether this is related to delayed diagnosis, differing tumor characteristics, or treatment inequalities.

Methods

The Cox model was applied to gastric adenocarcinoma cases diagnosed during 2004-2011 in American residents aged 20+ years in the Surveillance, Epidemiology, and End Results (SEER) program to determine the impact of rurality on mortality. Binary logistic regression was used to compare the odds of not receiving surgical treatment for localized tumors between rural and urban areas. It was also used to measure the association of rurality with stage at diagnosis (non-metastatic vs. metastatic).

Results

There was a significant association of rurality on 5-year mortality [HR 1.14 (1.09-1.20), p < 0.01]. No significant association was observed between rural-urban residency and stage at diagnosis, with an odds ratio (OR) of 0.95 (0.87-1.03), p = 0.21. The median time from diagnosis to any first-course treatment was one month for both rural and urban counties. Rural residents were far more likely not to receive surgical treatment for localized tumors than their urban counterparts [OR 1.70 (1.41-2.05), p < 0.01]. A greater percentage of rural inhabitants had cardia tumors as compared to urban ones, 39.8% vs. 33.8% respectively. Non-cardia tumors were far less likely not to receive surgical treatment (i.e., more likely to receive surgical treatment) than cardia tumors [OR 0.35 (0.30-0.41), p < 0.01].

Conclusions

Rurality is associated with worse gastric adenocarcinoma mortality. This may be due to a lesser probability of receiving surgical treatment for early-stage disease and differences in the primary site of the tumor between rural and urban counties, but not due to differences in stage at presentation. Future research should focus on improving health care access in rural communities.

## Introduction

Gastric cancer is one of the most prevalent cancers in the world. It represents 5.7% of new cancer cases and 8.2% of cancer-related deaths, making it the third most common cause of death from cancer [1-3]. In the United States, the American Cancer Society estimates 26,560 new cases and 11,180 deaths from gastric cancer in 2021 [4]. Adenocarcinomas comprise 90% of gastric cancers and tend to arise from atrophic gastritis, which may occur from *Helicobacter pylori* infection, antibodies to acid-secreting parietal cells, or surgical destruction of the antrum which releases gastrin [5,6]. Localized tumors have a much better prognosis than more advanced disease [7,8]. Additionally, the diagnosis and treatment are often complex and require a multifaceted approach. Hence, appropriate and timely management is essential for better patient outcomes.

Sociodemographic factors can significantly impact proper care. Lower educational levels and income, for instance, may result in a worse cancer prognosis [9]. A prior study highlighted that patients living in rural counties have worse survival in cervical, colorectal, lung, and prostate cancers than their urban counterparts [10]. However, there is a paucity of data with regards to the role of rurality in the care of patients with gastric cancer. The rural population is known to suffer from limited access to oncology providers, longer commute times, and a greater likelihood of being uninsured than their urban counterparts [11]. This trend is worrisome since one-fifth of the US population lives in rural areas. Our aim was to determine if rural inhabitation affects the mortality of patients with gastric adenocarcinoma. If such an association exists, we propose to ascertain whether this is related to delayed diagnosis, differing tumor characteristics, or treatment inequalities.

## Materials and methods

The Surveillance, Epidemiology, and End Results (SEER) program is well-known as a source of epidemiologic data on various malignancies [12]. Within SEER, we selected county attributes from the database “County Attributes - Total U.S., 1969-2018 Counties” for our study. These include the median household income and the percentage of people who received high school education and have ever smoked. Attributes from unknown counties were excluded. 

**Figure 1:**
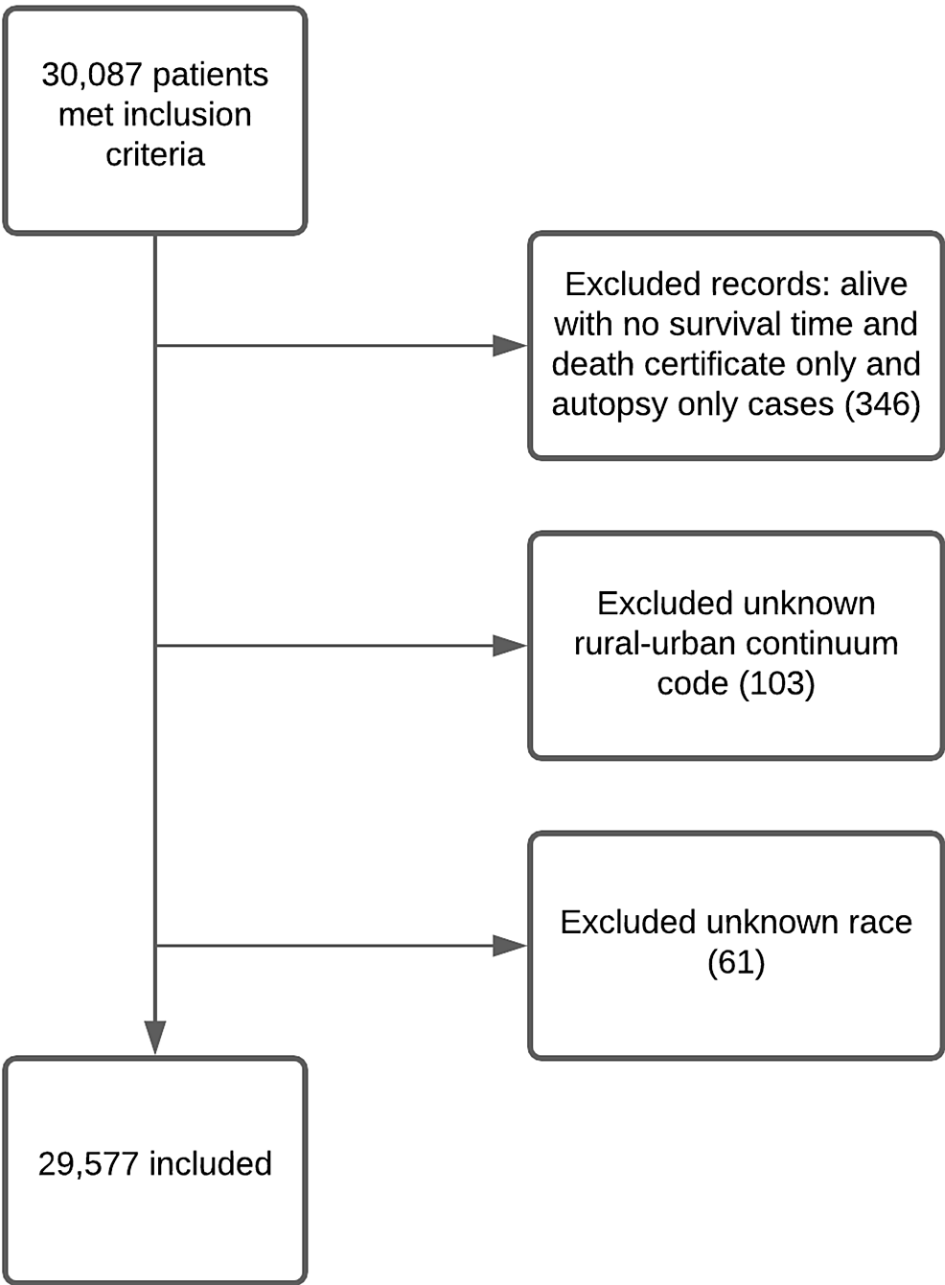
Flowchart of the study selection process.

We used the database “SEER Regs 18 Custom Data with Calculated Months Fields (since last birthday/from dx to treatment) and additional treatment fields, Nov 2018 Sub” to identify patients with gastric cancer, using site recode International Classification of Diseases for Oncology 3th edition (ICD-O-3) “stomach”. Patients selected were at least 20 years old and diagnosed between the years 2004-2011. Only cases with malignant behavior were selected. We identified those with gastric adenocarcinoma using the ICD-O-3 codes 8140-8147. We excluded patients with unknown Rural-Urban Continuum Code (RUCC), who were alive with no survival time, those with death certificate only or autopsy only cases, and those with unknown race (Figure 1). The study end date was December 2016 to allow for at least a 5-year follow-up for our patient cohort.

Demographic and clinical data included age at diagnosis, grade and primary site of the tumor, marital status, place of residence, race, sex, stage, and treatment. Treatment types include chemotherapy, radiation, and surgery. We categorized the place of residence as rural or urban. Patients were grouped using the RUCC, which was divided into metropolitan (RUCCs 1-3), non-metropolitan (RUCCs 4-7), and completely rural (RUCCs 8-9) areas as per the Office of Management and Budget (OMB). RUCCs 1-3 include a population ranged from less or more than 250,000. RUCCs 4-7 include 20,000 or more individuals adjacent to a metropolitan area. RUCCs 8-9 include completely rural or less than 2,500 urban population adjacent or not adjacent to a metro area. Consistent with other studies, RUCCs 1-3 were classified as urban and RUCCs 4-9 as rural [13,14].

Marital status was categorized into married (including common law) and not married, which included divorced, never married, separated, widowed, and unknowns. For tumor staging, SEER summary staging was used, and SEER reports this to be the most precise clinical and pathological documentation of the extent of the disease [15]. Surgical treatment included local tumor destruction/excision and gastrectomy. The primary site was categorized into cardia, non-cardia, overlapping lesions, and stomach not otherwise specified (NOS). Non-cardia tumors excluded cardia and overlapping lesions and included those in the antrum, body, fundus, greater and lesser curvature, and pylorus. SEER defines overlapping lesions as primary malignant neoplasms that overlap two or more contiguous sites.

We used the Pearson's Chi-square test to assess the associations between categorical variables, with the assumption of independence of observations and a relatively large sample size. Binary logistic regression was used to compare the odds of not receiving surgical treatment for localized tumors between rural and urban areas. It was also used to measure the association of rurality with the stage at diagnosis. The impact of RUCC on mortality was determined using Cox proportional hazards model. We adjusted for patient-specific data including age at diagnosis, grade and primary site of the tumor, marital status, place of residence, race, sex, and stage. SEER categorizes chemotherapy data as either “yes - patient had chemotherapy” or “no/unknown - no evidence of chemotherapy was found in the medical records examined”. Radiation therapy is categorized similarly. Thus, we created two models in our binary logistic regression and Cox proportional hazard models. Model 1 does not include treatment. Model 2 includes treatment and categorizes these variables as reported by SEER. All statistics were performed using the SEER*Stat software (National Cancer Institute, Bethesda, USA) and the Statistical Package for Social Sciences (SPSS), version 27.0 (IBM Corp., Armonk, USA) statistical package. The East Tennessee State University institutional review board (IRB) determined that this study neither meets the US FDA nor the Department of Health and Human Services' definition of human subjects research, thus it does not fall under the purview of the IRB.

## Results

Patient characteristics and epidemiology

**Figure 2:**
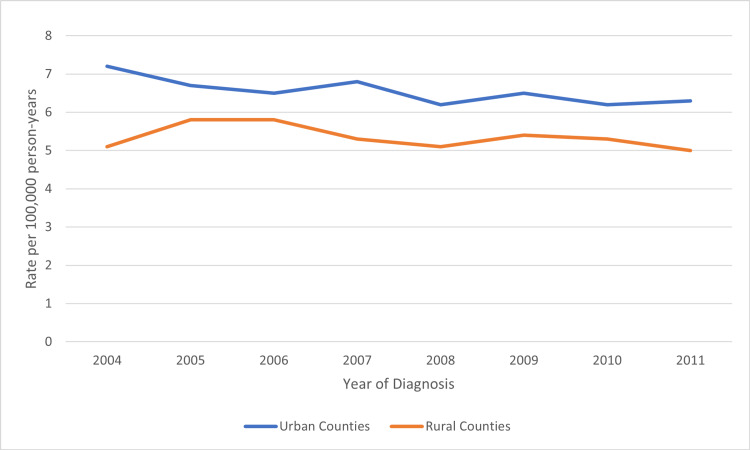
Age-Adjusted Incidence Rate of Gastric Adenocarcinoma, 2004-2011

A total of 29,577 cases of gastric adenocarcinoma were identified in the United States between 2004-2011. 26,437 (89.4%) patients were in urban counties (UC) and 3,140 (10.6%) in rural counties (RC). The age-adjusted incidence rate of gastric adenocarcinoma in UC was 6.5 per 100,000 person-years [95% confidence interval (CI) 6.4-6.6], and in RC it was 5.3 per 100,000 person-years (CI 5.1-5.5). It has remained stable in RC and UC, despite some fluctuation (Figure 2). The baseline characteristics of the 29,577 patients are shown in Table 1. In both rural and urban counties, most patients were white, married, and male.

**Table 1:** Baseline Characteristics of Gastric Adenocarcinoma Patients from 2004-2011 Percentages have been rounded and may not total 100. SEER: Surveillance, Epidemiology, and End Results

	Urban (n = 26,437)		Rural (n = 3140)	
Age at diagnosis (years)	69.7		69.7	
Race (n, %)				
White	14,431	54.6%	2,240	71.3%
American Indian/Alaska Native	94	0.4%	54	1.7%
Asian or Pacific Islander	4,033	15.3%	212	6.8%
Black	3,489	13.2%	435	13.9%
Hispanic	4,390	16.6%	199	6.3%
Grade (n, %)				
Well/Moderately Differentiated	8,776	33.2%	1,107	35.3%
Poorly/Un-differentiated	14,102	53.3%	1,614	51.4%
Unknown	3,559	13.5%	419	13.3%
Marital Status (n, %)				
Unmarried	10,442	39.5%	1,235	39.3%
Married	14,781	55.9%	1,775	56.5%
Unknown	1,214	4.6%	130	4.1%
Primary Site (n, %)				
Cardia	8,928	33.8%	1,249	39.8%
Non-cardia	12,123	45.9%	1,283	40.9%
Overlapping lesion	1,846	7.0%	151	4.8%
Stomach, not otherwise specified	3,540	13.4%	457	14.6%
Sex (n, %)				
Male	17,327	65.5%	2,170	69.1%
Female	9,110	34.5%	970	30.9%
SEER Stage (n, %)				
Localized Only	6,234	23.6%	847	27.0%
Regional by direct extension only	1,437	5.4%	185	5.9%
Regional lymph nodes involved only	3,408	12.9%	378	12.0%
Regional by both direct extension and lymph node involvement	3,463	13.1%	372	11.8%
Distant site(s)/node(s) involved	9,279	35.1%	1,093	34.8%
Unknown	2,616	9.9%	265	8.4%
Chemotherapy (n, %)				
Yes	15,275	57.8%	1,839	58.6%
None/Unknown	11,162	42.2%	1,301	41.4%
Radiation (n, %)				
Yes	6,563	24.8%	836	26.6%
None	280	1.1%	58	1.8%
None/Unknown	19,594	74.1%	2,246	71.5%
Surgery (n, %)				
Yes	12,164	46.0%	1,326	42.2%
None	14,147	53.5%	1,767	56.3%
Unknown	126	0.5%	47	1.5%

County-level attributes

County-level attributes are shown in Table 2. The population size reported is from the year 2004. The percentage of those with less than a high school education includes people aged 25 years and over for 2000. The median household income is from 2000. The percentage of ever smokers is from 2004-2007, and a person 18 years and older must have smoked at least 100 cigarettes by the time of the interview.

**Table 2:** County-Level Attributes

	Urban	Rural
Population size (2004)	73.2 million	8.7 million
% < High School Education (2000)	19.8%	24.1%
Median Household Income (2000)	$41,782	$32,072
Percentage of Ever Smokers (2004-2007)	45.0%	48.5%

Primary site

A cross-tabulation of the primary site and RUCC is shown in Table 3. It indicates that the observed count was higher than the expected count for cardia tumors in RC, while the inverse was true for UC. The observed count for non-cardia tumors was higher than the expected count in UC. Pearson's Chi-square indicated that the relationship between the primary site and RUCC was significant, *X*^2^ (3, N = 29,577) = 67.3, p < 0.01.

**Table 3:** Cross-Tabulation of Primary Site and Rural-Urban Continuum Code for Gastric Adenocarcinoma, 2004-2011 RUCC: Rural-Urban Continuum Code

			Rural-Urban Continuum Code		Total
			rural	urban	
Primary Site	Cardia	Count	1249	8928	10177
		Expected Count	1080.4	9096.6	10177.0
		% within RUCC	39.8%	33.8%	34.4%
	Non-cardia	Count	1283	12123	13406
		Expected Count	1423.2	11982.8	13406.0
		% within RUCC	40.9%	45.9%	45.3%
	Overlapping Lesion	Count	151	1846	1997
		Expected Count	212.0	1785.0	1997.0
		% within RUCC	4.8%	7.0%	6.8%
	Stomach, not otherwise specified	Count	457	3540	3997
		Expected Count	424.3	3572.7	3997.0
		% within RUCC	14.6%	13.4%	13.5%

Stage at diagnosis

To compare the stage at diagnosis between RC and UC, we divided cases into non-metastatic and metastatic and controlled for various sociodemographic factors (Table 4). Metastatic cases included those with the distant site or lymph node involvement. No significant association was observed between rural-urban residency and stage at diagnosis, with an odds ratio (OR) of 0.95 (0.87-1.03), p = 0.21.

**Table 4:** Association of Rural-Urban Residency with Stage at Diagnosis of Gastric Adenocarcinoma, Adjusted for Age at Diagnosis, Marital Status, Race, and Sex. RUCC: Rural-Urban Continuum Code

	Odds Ratio for Metastatic Disease	95% Confidence Interval	P value
Age	0.98	(0.98-0.98)	p < 0.01
Race			
White [reference]			
American Indian/Alaska Native	0.96	(0.66-1.38)	p = 0.81
Asian or Pacific Islander	0.72	(0.66-0.78)	p < 0.01
Black	0.96	(0.89-1.04)	p = 0.32
Hispanic	1.03	(0.96-1.11)	p = 0.45
RUCC			
Urban [reference]			
Rural	0.95	(0.87-1.03)	p = 0.21
Marital Status			
Unmarried [reference]			
Married	0.94	(0.89-0.99)	p = 0.03
Sex			
Female	0.97	(0.92-1.03)	p = 0.32

Surgical treatment for early stage

Table 5 describes the association of rural-urban residency on surgical treatment in localized gastric adenocarcinoma. We found that rural residents were far more likely not to receive surgical treatment for localized tumors than their urban counterparts [OR 1.70 (1.41-2.05), p < 0.01]. Non-cardia tumors, excluding overlapping lesions, were far less likely not to receive surgical treatment (i.e., more likely to receive surgical treatment) than cardia tumors [OR 0.35 (0.30-0.41), p < 0.01].

**Table 5:** Association of Rural-Urban Residency on Surgical Treatment in Localized Gastric Adenocarcinoma, Adjusted for Age at Diagnosis, Grade and Primary Site of the Tumor, Marital Status, Race, Sex, With And Without Treatment. RUCC: Rural-Urban Continuum Code

	Model 1 (not adjusted for treatment)			Model 2 (adjusted for treatment)		
	Odds Ratio for No Treatment	95% Confidence Interval	P-value	Odds Ratio for No Treatment	95% Confidence Interval	P-value
Age	1.06	(1.05-1.06)	p < 0.01	1.07	(1.06-1.07)	p < 0.01
Race						
White [reference]						
American Indian/Alaska Native	1.02	(0.39-2.66)	p = 0.96	1.03	(0.38-2.76)	p = 0.95
Asian or Pacific Islander	0.57	(0.46-0.70)	p < 0.01	0.59	(0.47-0.72)	p < 0.01
Black	1.59	(1.30-1.94)	p < 0.01	1.65	(1.34-2.03)	p < 0.01
Hispanic	1.26	(1.03-1.53)	p = 0.02	1.27	(1.04-1.54)	p = 0.02
RUCC						
Urban [reference]						
Rural	1.70	(1.41-2.05)	p < 0.01	1.65	(1.36-1.99)	p < 0.01
Grade						
Well/Moderately Differentiated [reference]						
Poorly/Un-differentiated	1.53	(1.35-1.74)	p < 0.01	1.42	(1.25-1.62)	p < 0.01
Marital Status						
Unmarried [reference]						
Married	0.64	(0.56-0.74)	p < 0.01	0.61	(0.53-0.71)	p < 0.01
Primary Site						
Cardia [reference]						
Non-cardia (excluding overlapping lesion)	0.35	(0.30-0.41)	p < 0.01	0.44	(0.38-0.51)	p < 0.01
Overlapping lesion	0.78	(0.58-1.04)	p = 0.09	0.92	(0.68-1.23)	p = 0.57
Sex						
Female	0.96	(0.83-1.11)	p = 0.60	0.54	(0.43-0.67)	p < 0.01
Chemotherapy						
Yes [reference]						
None/Unknown				0.55	(0.44-0.68)	p < 0.01
Radiation						
Yes [reference]						
None				1.89	(1.02-3.51)	p = 0.04
None/Unknown				0.54	(0.43-0.67)	p < 0.01

Survival and time to treatment, all stages

**Figure 3:**
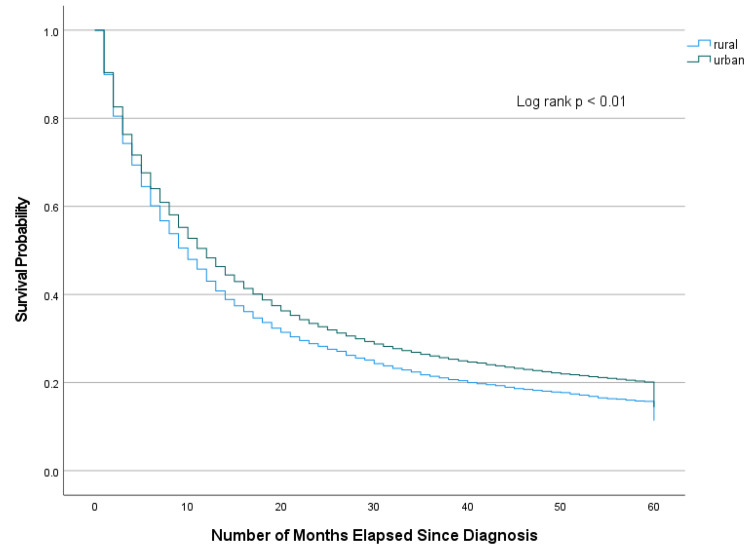
Five-Year Crude Survival of Gastric Adenocarcinoma Cases Diagnosed from 2004-2011

The median time from diagnosis of gastric adenocarcinoma to any first-course treatment, including chemotherapy, radiation, and surgery, was one month for both rural and urban counties. Table 6 depicts the median observed survival by SEER stage and cause-specific survival. The unadjusted median overall survival (OS) was 10.0 months in RC and 12.0 months in UC (log rank, p < 0.01). The median OS for all stages and the cause-specific survival were notably lower in RC compared to UC. The most drastic difference was noted in localized disease, of about 21 months. Kaplan-Meier plot for OS for all stages is depicted in Figure 3. Table 7 illustrates the association of rural-urban residency on the 5-year mortality. We found a significant association of rurality on mortality [HR 1.14 (1.09-1.20), p < 0.01].

**Table 6:** Median Survival of Gastric Adenocarcinoma Cases Diagnosed From 2004-2011 *Deaths due to all causes

	Urban	Rural
Median Survival, months*		
All stages	12	10
Localized Only	44	23
Regional by direct extension only	16	12
Regional lymph nodes involved only	25	23
Regional by both direct extension and lymph node involvement	16	14
Distant site(s)/node(s) involved	5	5
Median Cause-Specific Survival, months	14	12

**Table 7:** Association of Rural-Urban Residency on 5-Year Mortality in Gastric Adenocarcinoma, Adjusted for Age at Diagnosis, Grade and Primary Site of the Tumor, Marital Status, Race, Sex, Stage, With and Without Treatment. RUCC: Rural-Urban Continuum Code

5-Year Mortality						
	Model 1 (not adjusted for treatment)			Model 2 (adjusted for treatment)		
	Hazard Ratio	95% Confidence Interval	P-value	Hazard Ratio	95% Confidence Interval	P-value
Age	1.02	(1.02-1.02)	p < 0.01	1.01	(1.01-1.02)	p < 0.01
Race/ethnicity						
White [reference]						
American Indian/Alaska Native	1.21	(0.99-1.49)	p = 0.06	1.13	(0.92-1.38)	p = 0.23
Asian or Pacific Islander	0.78	(0.75-0.82)	p < 0.01	0.79	(0.75-0.83)	p < 0.01
Black	1.07	(1.02-1.12)	p < 0.01	1.04	(1.00-1.09)	p = 0.08
Hispanic	0.93	(0.89-0.97)	p < 0.01	0.90	(0.87-0.94)	p < 0.01
RUCC						
Urban [reference]						
Rural	1.14	(1.09-1.20)	p < 0.01	1.12	(1.07-1.18)	p < 0.01
Grade						
Well/Moderately Differentiated [reference]						
Poorly/Un-differentiated	1.24	(1.20-1.28)	p < 0.01	1.28	(1.24-1.32)	p < 0.01
Marital Status						
Unmarried [reference]						
Married	0.80	(0.77-0.82)	p < 0.01	0.87	(0.84-0.90)	p < 0.01
Primary Site						
Cardia [reference]						
Non-cardia (excluding overlapping lesion)	0.91	(0.88-0.94)	p < 0.01	0.99	(0.95-1.03)	p = 0.56
Overlapping lesion	1.15	(1.08-1.22)	p < 0.01	1.19	(1.12-1.26)	p < 0.01
Stomach, not otherwise specified	1.21	(1.15-1.27)	p < 0.01	1.17	(1.11-1.23)	p < 0.01
Sex						
Female	0.93	(0.90-0.96)	p < 0.01	0.92	(0.89-0.95)	p < 0.01
SEER Stage						
Localized Only [reference]						
Regional by direct extension only	1.61	(1.51-1.72)	p < 0.01	1.87	(1.75-2.00)	p < 0.01
Regional lymph nodes involved only	1.33	(1.27-1.40)	p < 0.01	1.83	(1.74-1.92)	p < 0.01
Regional by both direct extension and lymph node involvement	1.88	(1.79-1.98)	p < 0.01	2.77	(2.63-2.91)	p < 0.01
Distant site(s)/node(s) involved	4.48	(4.29-4.67)	p < 0.01	3.58	(3.41-3.75)	p < 0.01
Chemotherapy						
Yes [reference]						
None/Unknown				1.89	(1.82-1.96)	p < 0.01
Radiation						
Yes [reference]						
None				1.07	(0.93-1.23)	p = 0.32
None/Unknown				1.07	(1.03-1.11)	p < 0.01
Surgery						
Yes [reference]						
None				2.88	(2.77-2.99)	p < 0.01

## Discussion

Despite a lower incidence in rural counties compared to urban, gastric adenocarcinoma had worse median survival for almost all stages in rural counties, except metastatic for which it was equally poor at 5 months. Localized disease, which is highly curable, had the largest gap of 21 months, and rural patients were far less likely to receive surgical treatment compared to their urban counterparts. Rurality was independently correlated with worse mortality. We could not attribute rural-urban mortality differences with delayed diagnosis or treatment in the rural population.

A higher prevalence of comorbidities and opposing lifestyle behaviors may contribute to the increased mortality seen in rural counties. Rural inhabitants are more likely to report poor health, lack of physical activity, and chronic medical conditions such as obesity, hypertension, heart disease, diabetes, and depression [16]. We also observed a higher percentage of smokers in rural areas, roughly 48.5% compared to 45% in urban areas from 2004-2007. And the rural population is known to have less access to healthy food [17,18]. Ngoan et al. (2002) observed higher gastric cancer mortality in people consuming processed meat compared to those consuming green and yellow vegetables [19]. Comorbidities, however, do not fully account for the discrepancies in gastric adenocarcinoma mortality, since we noticed a higher median cause-specific survival in urban areas (i.e., rural patients were more likely to have gastric adenocarcinoma reported as the cause of death). Thus, we do not believe comorbidities in rural patients were severe enough to prevent them from receiving surgery since they would be expected to die from their comorbidities, similar to the belief Atkins et al. (2017) had in regard to the lower chance of rural patients receiving surgical treatment for stage I lung cancer [20].

Consistent with prior data [21], we found that the rural population tends to have lower attained education than the urban population does, with 24.1% having less than a high school education compared to 19.8%, respectively. Education is a crucial social determinant of health since it promotes improved awareness, reasoning abilities, and emotional self-control [22]. Thus, a higher educational level may increase one’s cognizance of disease and willingness to receive care. Rural patients suffer from more financial barriers as well, and this could further hinder the ability to seek treatment. For the year 2000, we observed that the median household income was $32,072 in rural counties compared to $41,782 in urban counties. These disparities adversely impact the care of patients with gastric cancer, and Fontana et al. (1998) demonstrated this when they noted worse gastric cancer prognosis in patients with lower educational levels and incomes [23].

Other concerns in the rural population include longer commute times, less contact with oncology providers, and a greater likelihood of being uninsured [11,24]. In 2019, 12% to 15% of oncologists worked in rural settings, and 20% of rural Americans lived more than 60 miles from an oncologist [11]. This may lead to difficulties in establishing sufficient patient-physician relationships if rural patients do not have adequate contact with their providers, since they may not be able to voice their concerns and a communication gap may exist. These issues markedly diminish care for rural patients with gastric cancer and lessen the opportunity for equal medical treatment compared with their urban counterparts.

We also observed a higher likelihood of having a cardia tumor in rural counties as compared to urban ones, around 39.8% vs. 33.8% respectively. The trend is consistent with that observed using the National Cancer Data Base and is worrisome [25]. The incidence of gastric cardia adenocarcinoma, along with other cancers of the gastroesophageal (GE) junction, has been rising worldwide and has been paralleled with increasing obesity [26]. Rural counties are known to have a higher prevalence of obesity than urban counties [27,28]. Unfortunately, we observed that cardia tumors were less likely to be treated surgically, which have demonstrated a worse prognosis in several studies [29-31]. Thus, the increased tendency of cardia tumors in the rural population adds further strain to their communities. We believe the impact of rurality on other cancers of the GE junction may be an interesting topic for future research.

Strengths and limitations

Our study is one of few that have examined the association of rural-urban residency on gastric cancer mortality and treatment. It is the largest one that has used SEER for this purpose, and SEER provided us with a diverse patient cohort with which we were able to account for several sociodemographic factors when examining rurality. Thus, our findings can be generalized to the larger US population.

However, our study has notable limitations. We did not examine insurance status since it was mostly available only for patients aged 65 years and older and including it would have greatly reduced the size of our patient cohort. We also were unable to directly adjust for household income, high school education, and smoking status in our analysis because this was not linked to individual patients with gastric cancer in SEER. We were limited in our chemotherapy and radiation analysis, since patients who did not undergo chemotherapy/radiation were grouped with patients who had uncertain chemotherapy/radiation status, since treatment is often received outside a hospital and it is possible that was not captured entirely. SEER reports chemotherapy and radiation are subject to incomplete information. Therefore, we created two separate models in our analyses, one which did and the other which did not adjust for treatment variables. Finally, the median time to treatment from the time of diagnosis was for any of the first-course treatments and not specific to one specific type of treatment, such as chemotherapy, radiation, and surgery.

## Conclusions

Rurality is associated with worse gastric adenocarcinoma mortality. We believe that this is largely due to a lesser probability of receiving surgical treatment for early-stage disease, in consideration of numerous sociodemographic challenges unique to the rural population including educational and financial barriers, higher prevalence of comorbidities, limited access to providers and treatments, and transportation obstacles. We observed differences in the primary site of the tumor between rural and urban counties which may contribute to this disparity, but we did not observe a difference in stage at presentation. Future research should be done on how to best target these challenges.

## References

[1] Bray F, Ferlay J, Soerjomataram I, Siegel RL, Torre LA, Jemal A (2018). Global cancer statistics 2018: GLOBOCAN estimates of incidence and mortality worldwide for 36 cancers in 185 countries. CA Cancer J Clin.

[2] Balakrishnan M, George R, Sharma A, Graham DY (2017). Changing trends in stomach cancer throughout the world. Curr Gastroenterol Rep.

[3] Torre LA, Bray F, Siegel RL, Ferlay J, Lortet-Tieulent J, Jemal A (2015). Global cancer statistics, 2012. CA Cancer J Clin.

[4] (2021). American Cancer Society. Cancer Facts & Figures. https://www.cancer.org/cancer/stomach-cancer/about/key-statistics.html.

[5] Kumar RK, Raj SS, Shankar EM, Ganapathy E, Ebrahim AS, Farooq SM (2013). Gastric carcinoma: a review on epidemiology, current surgical and chemotherapeutic options. Gastric carcinoma-new insights into current management. IntechOpen.

[6] Park YH, Kim N (2015). Review of atrophic gastritis and intestinal metaplasia as a premalignant lesion of gastric cancer. J Cancer Prev.

[7] Asplund J, Kauppila JH, Mattsson F, Lagergren J (2018). Survival trends in gastric adenocarcinoma: a population-based study in Sweden. Ann Surg Oncol.

[8] Van Cutsem E, Sagaert X, Topal B, Haustermans K, Prenen H (2016). Gastric cancer. Lancet Lond Engl.

[9] Wu CC, Hsu TW, Chang CM, Yu CH, Wang YF, Lee CC (2014). The effect of individual and neighborhood socioeconomic status on gastric cancer survival. PLoS One.

[10] Singh GK, Williams SD, Siahpush M, Mulhollen A (2011). Socioeconomic, rural-urban, and racial inequalities in us cancer mortality: part I-all cancers and lung cancer and part II-colorectal, prostate, breast, and cervical cancers. J Cancer Epidemiol.

[11] Levit LA, Byatt L, Lyss AP (2020). Closing the rural cancer care gap: three institutional approaches. JCO Oncol Pract.

[12] (2020). Surveillance Research Program, National Cancer Institute SEER*Stat software version 8.3.8. http://seer.cancer.gov/seerstat.

[13] Glynn ME, Keeton KA, Gaffney SH, Sahmel J (2018). Ambient asbestos fiber concentrations and long-term trends in pleural mesothelioma incidence between urban and rural areas in the United States (1973-2012). Risk Anal.

[14] Yu L, Sabatino SA, White MC (2019). Rural-urban and racial/ethnic disparities in invasive cervical cancer incidence in the United States, 2010-2014. Prev Chronic Dis.

[15] Ruhl JL, Callaghan C, Schussler N (eds.) (2021). Summary Stage 2018 Codes and Coding Instructions.

[16] Shaw KM, Theis KA, Self-Brown S, Roblin DW, Barker L (2016). Chronic disease disparities by county economic status and metropolitan classification, behavioral risk factor surveillance system, 2013. Prev Chronic Dis.

[17] Campbell EA, Shapiro MJ, Welsh C, Bleich SN, Cobb LK, Gittelsohn J (2017). Healthy food availability among food sources in rural maryland counties. J Hunger Environ Nutr.

[18] Lutfiyya MN, Chang LF, Lipsky MS (2012). A cross-sectional study of US rural adults' consumption of fruits and vegetables: do they consume at least five servings daily?. BMC Public Health.

[19] Ngoan LT, Mizoue T, Fujino Y, Tokui N, Yoshimura T (2002). Dietary factors and stomach cancer mortality. Br J Cancer.

[20] Atkins GT, Kim T, Munson J (2017). Residence in rural areas of the united states and lung cancer mortality. Disease incidence, treatment disparities, and stage-specific survival. Ann Am Thorac Soc.

[21] Marre A (2017). Rural Education at a Glance.

[22] Hahn RA, Truman BI (2015). Education improves public health and promotes health equity. Int J Health Serv.

[23] Fontana V, Decensi A, Orengo MA, Parodi S, Torrisi R, Puntoni R (1998). Socioeconomic status and survival of gastric cancer patients. Eur J Cancer Oxf Engl.

[24] Henley SJ, Jemal A (2018). Rural cancer control: bridging the chasm in geographic health inequity. Cancer Epidemiol Biomarkers Prev.

[25] Rana N, Gosain R, Lemini R (2020). Socio-demographic disparities in gastric adenocarcinoma: a population-based study. Cancers (Basel).

[26] O'Doherty MG, Freedman ND, Hollenbeck AR, Schatzkin A, Abnet CC (2012). A prospective cohort study of obesity and risk of oesophageal and gastric adenocarcinoma in the NIH-AARP Diet and Health Study. Gut.

[27] Befort CA, Nazir N, Perri MG (2012). Prevalence of obesity among adults from rural and urban areas of the United States: findings from NHANES (2005-2008). J Rural Health.

[28] (2021). CDC: More obesity in U.S. rural counties than in urban counties | CDC Online Newsroom | CDC. Published April 11. https://www.cdc.gov/media/releases/2018/s0614-obesity-rates.html.

[29] Chen WY, Cheng HC, Wang JD, Sheu BS (2013). Factors that affect life expectancy of patients with gastric adenocarcinoma. Clin Gastroenterol Hepatol.

[30] Ghidini M, Donida BM, Totaro L (2020). Prognostic factors associated with survival in a large cohort of gastric cancer patients resected over a decade at a single Italian center: the Cremona experience. Clin Transl Oncol.

[31] Kim YJ, Chung WC, Cho IH, Kim J, Kim S (2019). Prognostic effect of different etiologies in patients with gastric cardia cancer. Medicine (Baltimore).

